# Musculoskeletal ultrasound in the Differential Diagnosis of Gouty Arthritis and Rheumatoid Arthritis

**DOI:** 10.12669/pjms.36.5.2716

**Published:** 2020

**Authors:** Shao-wei Xue, Yu-kun Luo, Yu-rong Zhao, Zi-yu Jiao

**Affiliations:** 1 Shao-wei Xue, Department of Ultrasound, The First Medical Center of Chinese PLA General Hospital, Beijing 100853, P. R. China; 2 Yu-kun Luo, Department of Ultrasound, The First Medical Center of Chinese PLA General Hospital, Beijing 100853, P. R. China; 3 Yu-rong Zhao, Department of Ultrasound, The First Medical Center of Chinese PLA General Hospital, Beijing 100853, P. R. China; 4 Zi-yu Jiao, Department of Ultrasound, The First Medical Center of Chinese PLA General Hospital, Beijing 100853, P. R. China

**Keywords:** Gouty Arthritis, Musculoskeletal ultrasound, Rheumatoid Arthritis

## Abstract

**Objective:**

To explore the role of musculoskeletal ultrasound (MSUS) in the differential diagnosis of gouty arthritis (GA) and rheumatoid arthritis (RA) and to analyze the ultrasound imaging features of the two diseases.

**Methods:**

A retrospective study was carried out. A total of 66 patients who had been admitted to The First Medical Center of Chinese PLA General Hospital from May 2018 to March 2019 were enrolled. Among them, 34 patients were diagnosed with RA and were included in the RA group; 32 patients were diagnosed with gouty arthritis and were included in the GA group. The imaging features of musculoskeletal ultrasound were compared between the two groups of patients.

**Results:**

A total of 34 patients were included in the RA group, including 17 males and 17 females. A total of 32 patients were included in the GA group, including 14 males and 18 females. There were no significant differences in gender composition, age, and duration of disease between the two groups (*P*>0.05). In the RA group, there were joint bone erosions with a clear boundary in seven cases and with a blurred boundary in 27 cases; synovial hyperplasia was observed in 27 cases, and point-like hyperechoic masses were observed in four cases. In the GA group, there were joint bone erosions with a clear boundary in 27 cases and with a blurred boundary in five cases; synovial hyperplasia was observed in four cases, tophus was observed in 23 cases, point-like hyperechoic masses were observed in 27 cases, and the tram-track sign was observed in 23 cases. The differences in bone erosion boundaries (c^2^=26.854, *P*<0.01), synovial hyperplasia (c^2^=29.631, *P*<0.01), tophus (*P*<0.01), point-like hyperechoic mass (c^2^=33.095, *P*<0.01), and tram-track sign (*P*<0.01) were statistically significant between the two groups of patients. In the RA group, blood flow signaling was Grade 0 in one case, Grade-I in five cases, Grade-II in 14 cases, and Grade-III in 14 cases. In the GA group, blood flow signaling was Grade 0 in 26 cases, Grade-I in three cases, Grade-II in three cases, and Grade-III in zero cases. The difference in the synovial blood flow signaling between the two groups of patients was statistically significant (c^2^=34.323, *P*<0.01).

**Conclusions:**

MSUS has certain diagnostic value in the differentiation of GA and RA. Moreover, the two conditions have their own ultrasound imaging features.

## INTRODUCTION

Rheumatoid arthritis (RA) is a common chronic inflammatory disease mainly involving the small joints with an incidence of approximately 1.5% to 2%.^1^ Gouty arthritis (GA) is aseptic joint inflammation caused by the precipitation and deposition of monosodium urate (MSU) in the joint fluid. Its most typical clinical symptom is acute onset pain. Both conditions can involve the small joints and result in tissue stiffness and gradual loss of function in the patients’ joints as they progress. Therefore, early diagnosis and treatment have become the consensus of clinicians. With the development of ultrasound technology, musculoskeletal ultrasound (MSUS) has shown great advantages in capturing images of bones and soft tissues compared with conventional examinations, such as X-ray, CT, and MR. This study was designed to explore the role of MSUS in the differential diagnosis of RA and GA and to investigate the ultrasound imaging features of these two conditions.

## METHODS

### General data

A total of 66 patients who had been admitted to The First Medical Center of Chinese PLA General Hospital from May 2018 to March 2019 were retrospectively selected. Among them, 34 patients were diagnosed with rheumatoid arthritis and were included in the RA group; 32 patients were diagnosed with gouty arthritis and were included in the GA group.

### Inclusion criteria

1. Age > 18 years old.
2. Patients in the RA group should be diagnosed with RA and meet the 2010 American/European rheumatoid arthritis classification criteria^2^; and
3. Patients in the GA group should meet the clinical manifestations of gout^3^ and be confirmed with hyperuricemia.

### Exclusion criteria

1. Age < 18 years old.
2. History of joint trauma or surgery.
3. Congenital joint deformity or disease; and
4. Arthritis other than RA and GA, such as psoriatic arthritis.

### Ethical approval

The study was approved by the Institutional Ethics Committee of The First Medical Center of Chinese PLA General Hospital at February 7, 2020 and written informed consent was obtained from all participants.

The general data of patients in the two groups were checked for balance. There was no significant difference between the two groups, and they were comparable.

### Examination methods

A Philips IU22 Ultrasound Machine with a 6-16 MHz high frequency probe was used to check the metacarpal and interphalangeal joints of both groups of patients. A combination of longitudinal and transverse sections was used to examine the medial, lateral, dorsal, palm, and other parts of the patients’ joints. The images were saved, and the imaging features inside the joints were carefully observed and recorded. All of these operations and imaging analyses were performed by the same senior experienced ultrasound physician who had received dedicated MSUS training.

### Statistical indicators

The general information on patients in the two groups, including gender, age, and duration of condition, were recorded. The ultrasound imaging features of the metacarpophalangeal joints of the patients, including clarity of bone erosion boundary, synovial hyperplasia, joint tophus, point-like hyperechoic mass, tram-track sign, and blood flow signal, were also recorded.

*Tophus:*A cluster of high and low echoes in the ultrasound images that is predominated by high echoes and is often accompanied by acoustic shadowing;

*Point-like hyperechoic mass:*Caused by the deposition of the urate crystals in the joints;

Tram-track sign: The urate crystals precipitate from the joint fluid and deposit on the surface of articular cartilage. Two hyperechoic lines are formed by the urate crystals on the articular surface.

Blood flow signaling:

A semi quantitative grading was used to score the signal. No blood flow signal in the synovial membrane is 0 points; a single blood flow signal in the synovial membrane is one point; branches of blood flow signals in the synovial membrane, which is smaller than 1/2 of the area of the synovial membrane, is two points; and branches of blood flow signals in the synovial membrane, which is larger than 1/2 of the area of the synovial membrane, is three points.

### Statistical methods

Statistical analysis was performed using SPSS 21.0 software. All data were expressed as the mean ± standard deviation (χ ± s), and the test level P value was 0.05. The normality analysis was first performed on the measurement data between groups, and the independent sample t test was used for measurement data that met the normality criteria. The chi-square test was used for comparison of count data.

## RESULTS

A total of 34 patients were included in the RA group, including 17 males and 17 females, with an average age of 45.764 ± 6.005 years and an average duration of disease of 4.117 ± 1.492 years. A total of 32 patients were included in the GA group, including 14 males and 18 females, with an average age of 46.062 ± 6.354 years and an average duration of disease of 3.843 ± 1.885 years. There were no statistically significant differences in the sex composition, age, and duration of disease between the two groups (*P*>0.05) (Table-I).

**Table-I:** General data of two groups of patients.

	No. of cases	Age (years old)	Duration of disease (years)	Gender (male/female)
RA group	34	45.764±6.005	4.117±1.492	17/17
GA group	32	46.062±6.354	3.843±1.885	14/18
Statistics		t=-0.196	t=0.656	c^2^=0.259
P value		>0.05	>0.05	>0.05

In the RA group, there were joint bone erosions with clear boundary in seven cases and with blurred boundary in 27 cases; synovial hyperplasia in 27 cases; tophus in 0 cases; point-like hyperechoic mass in four cases; and tram-track sign in 0 cases. In the GA group, there were joint bone erosions with clear boundary in 27 cases and with blurred boundary in five cases; synovial hyperplasia in four cases; tophus in 23 cases; point-like hyperechoic mass in 27 cases; and tram-track sign in 23 cases. The differences in bone erosion boundaries (c^2^=26.854, *P*<0.01), synovial hyperplasia (c^2^=29.631, *P*<0.01), tophus (*P*<0.01), point-like hyperechoic mass (c^2^=33.095, *P*<0.01), and tram-track sign (*P*<0.01) were statistically significant between the two groups of patients (Table-II).

**Table-II:** Ultrasound imaging features inside joints of two groups of patients.

	No. of cases	Bone erosion boundary		Synovial hyperplasia		Tophus		Point-like hyperechoic mass		Tram-track sign	

		Clear	Blurred	Yes	No	Yes	No	Yes	No	Yes	No
RA group	34	7	27	27	7	0	34	4	28	0	34
GA group	32	27	5	4	28	23	9	27	5	23	9
c^2^		26.854		29.631				33.095			
P value		<0.01		<0.01		<0.01*		<0.01		<0.01*	

***Note:***

* denotes Fisher’s exact test.

In the RA group, the blood flow signal was Grade 0 in one case, Grade-I in five cases, Grade-II in 14 cases, and Grade-III in 14 cases. In the GA group, the blood flow signal was Grade 0 in 26 cases, Grade-I in three cases, Grade-II in three cases, and Grade-III in zero cases. The difference in the synovial blood flow signal between the two groups of patients was statistically significant (c^2^=34.323, *P*<0.01).

## DISCUSSION

Rheumatoid arthritis is a common chronic inflammatory disease that mainly involves the joints. It can also cause arthritis symptoms, such as rheumatoid nodules, vasculitis, and interstitial lung disease.^4^ Typical symptoms include paroxysmal joint swelling and tenderness, joint stiffness in the morning, and laboratory abnormalities, such as elevated C-reactive protein and erythrocyte sedimentation rate. With inflammatory infiltration, it can cause joint swelling, which in turn leads to limited joint mobility and function. As a result, the patients suffer from compromised ability to work and perform daily activities, which may increase the burden on the society and country.^5^ Gouty arthritis develops as monosodium urate deposits in the joints, which induces the oxidative stress response of synovial cells, inflammatory cell infiltration, and cartilage damage.^6^-^9^ Typical symptoms of GA are joint swelling and pain in an acute attack. After years of recurrent episodes, it can progress to chronic GA, leading to joint stiffness, pain, and injury.^3^,^10^ The progression of both types of inflammatory conditions can lead to the loss of joint function in patients. Therefore, early diagnosis and treatment have become the consensus of clinicians.

**Table-III:** Synovial blood flow signal of two groups of patients.

	Grading of the blood flow signal			

	Grade 0	Grade-I	Grade-II	Grade-III
RA group	1	5	14	14
GA group	26	3	3	0
c^2^	34.323			
P value	<0.01			

Conventional imaging tests are X-rays, CT, and MR. Erosion and deformation of joints, which are more sensitive to X-rays, often appear years after the disease progresses. Moreover, X-ray is inferior to ultrasound in terms of sensitivity and specificity to the changes in soft tissues.^11^ However, CT remains inadequate in the diagnosis of changes in soft tissues and produces many radiation exposures.^12^ In the diagnosis of synovitis, MR does not have advantages over ultrasound, and it has the disadvantages of long examination time and high cost, which make it difficult to apply to the majority of the population.^13^ Ultrasonography is relatively convenient with fewer contraindications and lower costs, while allowing the examiner to communicate with patients. In the diagnosis of muscle and soft tissue abnormalities, the reliability and strengths of ultrasonography have been widely recognized.^14^-^16^

In this study, it was found that GA and RA had their own specific ultrasound imaging manifestations. When monosodium urate is deposited on the surface of the synovial membrane, it usually manifests as a point-like hyperechoic mass under ultrasound. As the crystal accumulation grows, tophus images with mixed high and low echoes are formed. As a result, tophus is generally considered to be a specific manifestation of GA.^17^-^19^ Meanwhile, since the bone erosions in GA is mostly caused by tophus, the boundary of the bone erosion area has no proliferative blood flow signal, making the boundary clear in GA. Due to the infiltration of inflammatory cells in the joint cavity, as well as synovial hyperplasia, the bone erosion area in RA is often accompanied by the proliferative blood flow signal and synovial hyperplasia. As a result, the bone erosion boundary is mostly blurred. Tram-track sign refers to two high-echo bright lines formed on the ultrasound images, with high sensitivity and specificity, as the monosodium urate is deposited on the joint surface over a long period of time.^19^,^20^

## CONCLUSION

In summary, the authors believes that MSUS has certain diagnostic value in the differentiation of GA and RA. Moreover, the two conditions have their own ultrasound imaging features.

### Authors’ Contributions

**SWX** **and** **YKL** designed this study and prepared this manuscript.

**YRZ** **and** **ZYJ** collected and analyzed clinical data and significantly revised this manuscript.

**SWX and** **YRZ** contributed this manuscript equally.

**YKL** is responsible and accountable for the accuracy or integrity of the study.
